# A 2-year longitudinal follow-up of quantitative assessment neck tics in Tourette’s syndrome

**DOI:** 10.1371/journal.pone.0261560

**Published:** 2021-12-30

**Authors:** Yosuke Eriguchi, Xiaoxue Gu, Naoto Aoki, Maiko Nonaka, Ryunosuke Goto, Hitoshi Kuwabara, Yukiko Kano, Kiyoto Kasai

**Affiliations:** 1 Department of Child Neuropsychiatry, Graduate School of Medicine, The University of Tokyo, Tokyo, Japan; 2 Division of Clinical Psychology, Graduate School of Education, The University of Tokyo, Tokyo, Japan; 3 Department of Neuropsychiatry, Sakura Hospital, Aomori, Japan; 4 Department of Pediatrics, The University of Tokyo Hospital, Tokyo, Japan; 5 Department of Psychiatry, Faculty of Medicine, Saitama Medical University, Saitama, Japan; 6 Department of Neuropsychiatry, Graduate School of Medicine, The University of Tokyo, Tokyo, Japan; Sohag University Faculty of Medicine, EGYPT

## Abstract

**Background:**

Neck motor tics in Tourette’s syndrome can cause severe neck complications. Although addressed in a few longitudinal studies, the clinical course of Tourette’s syndrome has not been quantitatively assessed. We had previously developed a method for quantifying the angular movements of neck tics using a compact gyroscope. Here, we present a follow-up study aimed at elucidating the clinical course of neck tics at both the group and individual levels.

**Methods:**

Eleven patients with Tourette’s syndrome from our previous study participated in the present study, and their neck tics were recorded during a 5-min observation period. The severity of neck symptoms was assessed using the Yale Global Tic Severity Scale. The peak angular velocities and accelerations, tic counts, and severity scores in our previous study (baseline) and the present study (2-year follow-up) were compared at the group and individual levels. The individual level consistency between baseline and follow-up were calculated using intra-class correlation coefficients (ICCs, one-way random, single measure).

**Results:**

At the group level, no significant change was observed between baseline and follow-up. At the individual level, angular velocity (ICC 0.73) and YGTSS scores (ICC 0.75) had substantial consistency over the two time points, and angular acceleration (ICC 0.59) and tic counts (ICC 0.69) had moderate consistency.

**Conclusions:**

The intensity and frequency of neck tics did not change over time. Therefore, quantification of angular neck motor tics will aid in identifying patients with neck tics at high risk for severe neck complications.

## Introduction

Tourette’s syndrome (TS) is a childhood-onset neurodevelopmental disorder characterized by motor and vocal tics. Tics are sudden, rapid, recurrent, and nonrhythmic movements or vocalizations. In some cases, TS follows a waxing and waning course, with one tic appearing and being replaced by another; however, in most cases, multiple tics are present concomitantly. Tics are sometimes treatment-refractory, but often decline in severity during adolescence [1–3].

Among TS motor tics, neck tics are some of the most common with a prevalence of around 60% among patients with TS [4, 5]. Furthermore, neck tics are potentially most damaging. Neck tics can lead to severe neck complications, including cervical myelopathy and vertebral artery dissection [6–9]. Despite the adverse effects of neck tics, their clinical course has yet to be investigated. Although previous longitudinal studies clinically assessed tic symptoms based on accounts of patients or observations by physicians, no quantitative longitudinal study has been conducted to date. A recent Swedish register-based study has revealed an increased risk of cervical spine disorders and related neurological complications among patients with TS or chronic tic disorder [10], further necessitating the understanding of the course of neck tics on the individual level.

We previously quantified the angular movement of neck tics using a compact gyroscope in order to determine the pathophysiological causes of severe neck complications. We recorded neck movements and assessed them in three orthogonal planes (yaw, pitch, roll). We found that neck tics in patients with TS had higher angular velocities and accelerations than did voluntary neck movements in healthy individuals.

In the present study, we conducted follow-up experiments on the TS patients from our previous study after a 2-year interval [11]. Our goal was to quantitatively elucidate the clinical course of neck tics at both the individual and group level. Angular velocity, angular acceleration, and the number of neck tics were recorded. As treatment refractory patients consistently manifest intense and frequent tics, we hypothesized that the intensity of neck tics would not change drastically over time despite the fluctuation of tic symptoms; consequently, a one-time measurement of angular movements could be valuable to identify TS patients at high risk for severe neck complications.

## Materials and methods

### Ethics statement

The purpose of this study was explained to all study participants, and all study participants subsequently provided written informed consent. For children, both the child and his or her parents provided written informed consent. The study was approved by the Ethics Committee of The University of Tokyo Hospital (No. 11504).

### Study participants

Patients with TS who had participated in our previous study [11] were potential candidates for the present study. In our previous study, we had screened the medical records of patients who had visited our clinic for TS and related disorders between September 2018 and June 2019. Among 182 patients with motor tic disorder aged more than 6 years (TS: 174; chronic motor tic disorder 7; transient motor tic disorder: 1), 39 patients with TS and two with chronic motor tic disorder who presented with neck tics during the previous study period had been recruited. Because 12 patients with TS and two with chronic motor tic disorder had declined participation, and five participants did not present with neck tics during previous experiments, the final study cohort comprised 22 TS patients (21 male and one female). Y.E. ascertained the time stamps of the patients’ motor tics according to the IMU output data using video streams. The patients’ neck tic movements were manually classified for each of the three planes (yaw, pitch, and roll). These participants were eligible for the present study if they had experimentally verified neck tics between October 2020 and June 2021 and had made regular follow-up visits to our clinic for TS and related disorders.

### Experimental setting and recording

The experimental procedures in sections 2.3 and 2.4 are the same as those described in our previous study [11]. Diagnoses were confirmed using Diagnostic and Statistical Manual of Mental Disorders, Fifth Edition [12]. The presence and severity of tic symptoms were evaluated using the Yale Global Tic Severity Scale (YGTSS) [13]. Five characteristics of motor and vocal tics (number, frequency, intensity, complexity, and interference of tics) were evaluated separately using ordinal scales. The total tic score was obtained by summation of the individual scores (0–50) and the YGTSS score (0–100). Comorbidities, medication histories, and histories of deep brain stimulation surgery were investigated.

During the entire observation procedure, participants were allowed to talk or play portable video games if necessary, to provoke neck tics. All participants were seated on folding chairs to minimize rotational body movements. An inertial measurement unit (IMU) (LPMS-B2 series; LP-RESEARCH Inc., Tokyo, Japan) was attached to the forehead of each participant using an LPMS-B2 holder strap (LP-RESEARCH Inc.). The IMU (size: 39 × 39 × 8 mm; weight, 12 g) is a wireless nine-axis motion sensor with a data transmission rate of 400 Hz. It includes a three-axis accelerometer, an a-axis gyroscope (Euler angles), and a three-axis magnetometer that outputs the acceleration, angular velocity, and magnetic field, respectively. The output data were transmitted to a laptop via a Bluetooth connection. A video camera was placed at a distance of 1–2 m from the participant to obtain a clear view of her/his upper body. Participants were instructed not to suppress their tics. Their head, neck, and shoulder movements, along with the corresponding IMU data, were recorded over a 5-min period using a camera connected to a laptop. The experiments were carried out in consultation rooms at the University of Tokyo hospital.

### Data processing

#### Classification of movements

**Fig 1:**
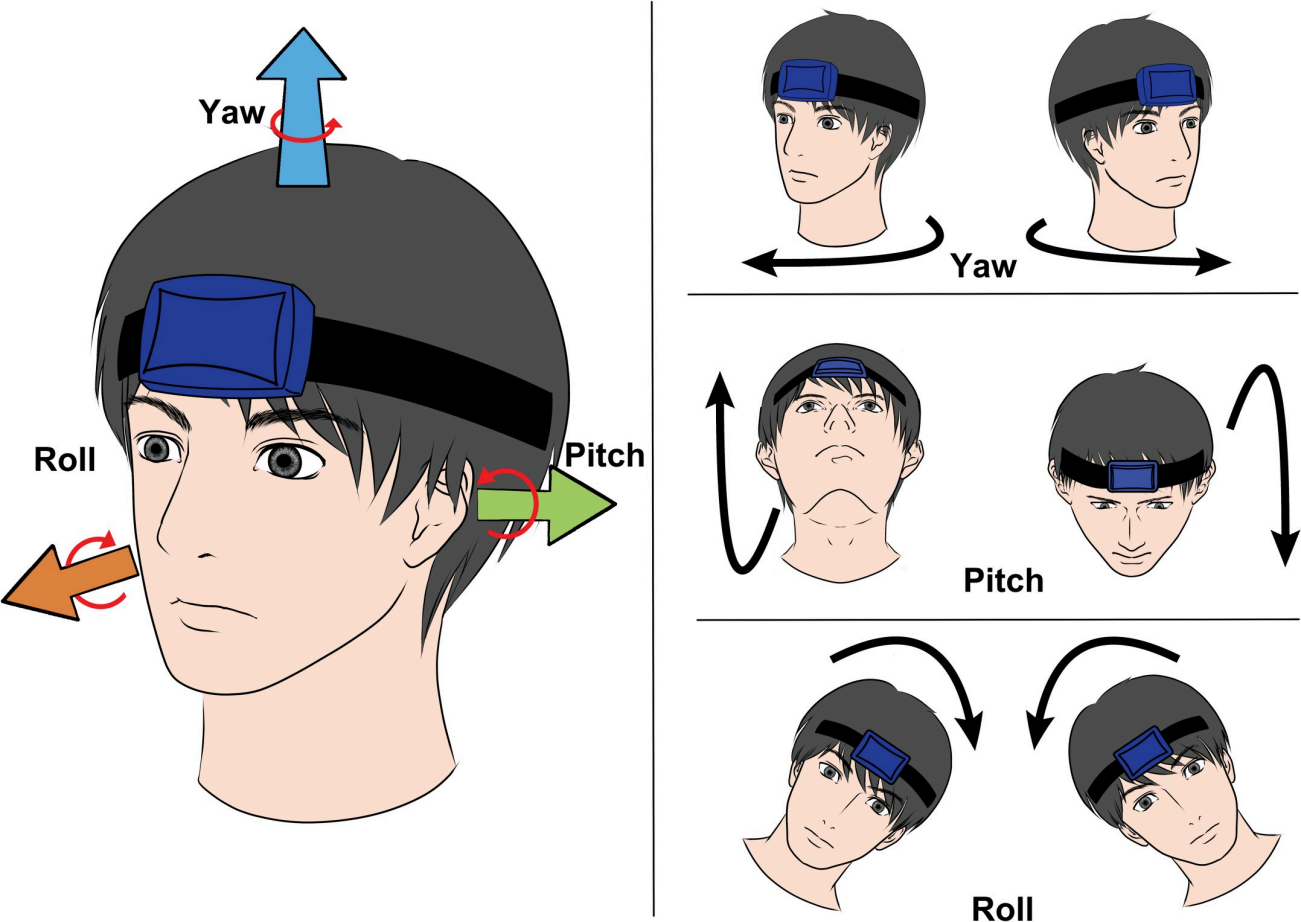
Diagram showing neck movements in three orthogonal planes.

Two authors (Y. E. and X. G.) ascertained the time stamps of the patients’ neck tics according to the IMU output data using the video streams. The neck tic movements were manually classified for each of the three planes (yaw, pitch, and roll, Fig 1). The tics occurring during the recording session were counted. Tic counting procedures were in accordance with those of previous studies [14, 15]. When the participant executed multiple repetitions of a tic movement in a single bout of tics, each tic was counted individually. All neck tics were counted regardless of simultaneous activity in other body regions.

#### Calculation of peak angular velocity and acceleration

Calculations were performed using R software for Windows (version 3.6.1). Using the RSpinCalc package [16], three-axis gyroscopic Euler angle data were converted into a quaternion for representation of angular velocity as scalars [17]. In addition, three-axis gyroscopic data were differentiated and converted into a quaternion for representation of angular acceleration as scalars. The maximum angular velocities and angular accelerations of neck tics were calculated.

### Statistical analyses

Two sets of data were compared: the baseline data collected in our previous study (September 2018 to June 2019) and the follow-up data obtained in the present study (October 2020 to April 2021). Before the main analyses, baseline data were compared between those who participated this study and those who only participated in our previous study. The number of patients with neck tics in each of the three rotational planes was compared between the two time points (i.e., baseline and follow-up) using the McNemar test. P-values equal to or less than 0.05 was considered statistically significant.

Normality of distribution was checked with Shapiro–Wilk test and confirmed by visualization using histograms. If the data were normally distributed, a paired t-test was used. Otherwise, Wilcoxon signed-rank test was used. To interpret the clinical course of neck tics at the group level, angular velocities, angular accelerations, tic counts, and YGTSS scores were compared between baseline and follow-up using the paired t test. The intra-class correlation coefficients (ICCs, one-way random, single measure) [18] were calculated to evaluate the individual level consistency between baseline and follow-up data. For interpretation of ICC values, the following ICC interpretation scale [19] was exploited: slight (0.00 to 0.20), fair (0.21 to 0.40), moderate (0.41 to 0.60), substantial (0.61 to 0.80), and almost perfect (0.81 to 1.00). The 95%-confidence interval (95% CI) and P-value of the ICC was computed as well. All data analyses were performed using R statistical software (version 3.6.1). Data are presented as mean ± standard deviation if the assumption of normality was not rejected. Otherwise, data are presented as median and interquartile range (IQR).

## Results

Nine of the 22 patients (21 male and one female) from our previous study had been transferred to other clinics, or discontinued regular follow-ups at our clinic, and two refused to participate further. Therefore, the data of 11 patients (10 male and one female, age: median, 22 [IQR: 14–24];) were analyzed. Sex ratio (P = 1.0), age (22 [24–24] vs 20 [11–30.5]) (P = 0.77, and YGTSS score (70 [59.5–80] vs 56 [38–73]) (P = 0.34) of those who participated in the present study did not differ from those who only participated in our previous study. Although angular velocity did not differ (7.22 [6.12–11.47] vs. 7.49 [3.95–8.68]) (P = 0.48), angular acceleration (1135.77 [613.76–1326.03] vs. 388.90 [194.86–564.81]) (P = 0.028) and tic count (17 [12–37.5] vs. 10 [6.5–12]) (P = 0.0067) of those who participated in this study were higher than those of individuals who only participated in our previous study. The interval between baseline and follow-up experiment is 711.00 ± 64.89 days.

One of the participants of the present study had undergone deep brain stimulation surgery. Attention deficit hyperactivity disorder, obsessive compulsive disorder, depression were noted in five (45.5%), four (36.4%), and five (45.5%) participants, respectively. Nine (81.8%) participants continued to take medications at the time of our experiments. One participant had a history of medication use, but he/she did not continue use at the time of our experiment. One participant did not have a history of medication use (S1 Table).

The presence or absence of tics in each rotational plane did not differ significantly between baseline and follow-up (yaw: P = 0.66; pitch: P = 0.32; roll: P = 0.56).

At the group level, the normality of distribution was evaluated using Shapiro-Wilk test and visual check of the histograms for baseline and follow-up data of angular velocity, angular acceleration, tic counts, and YGTSS score. Although Shapiro-Wilk test did not reject the null hypothesis, but histograms were apparently asymmetrical. Therefore, Wilcoxon signed-rank test was adopted.

**Fig 2:**
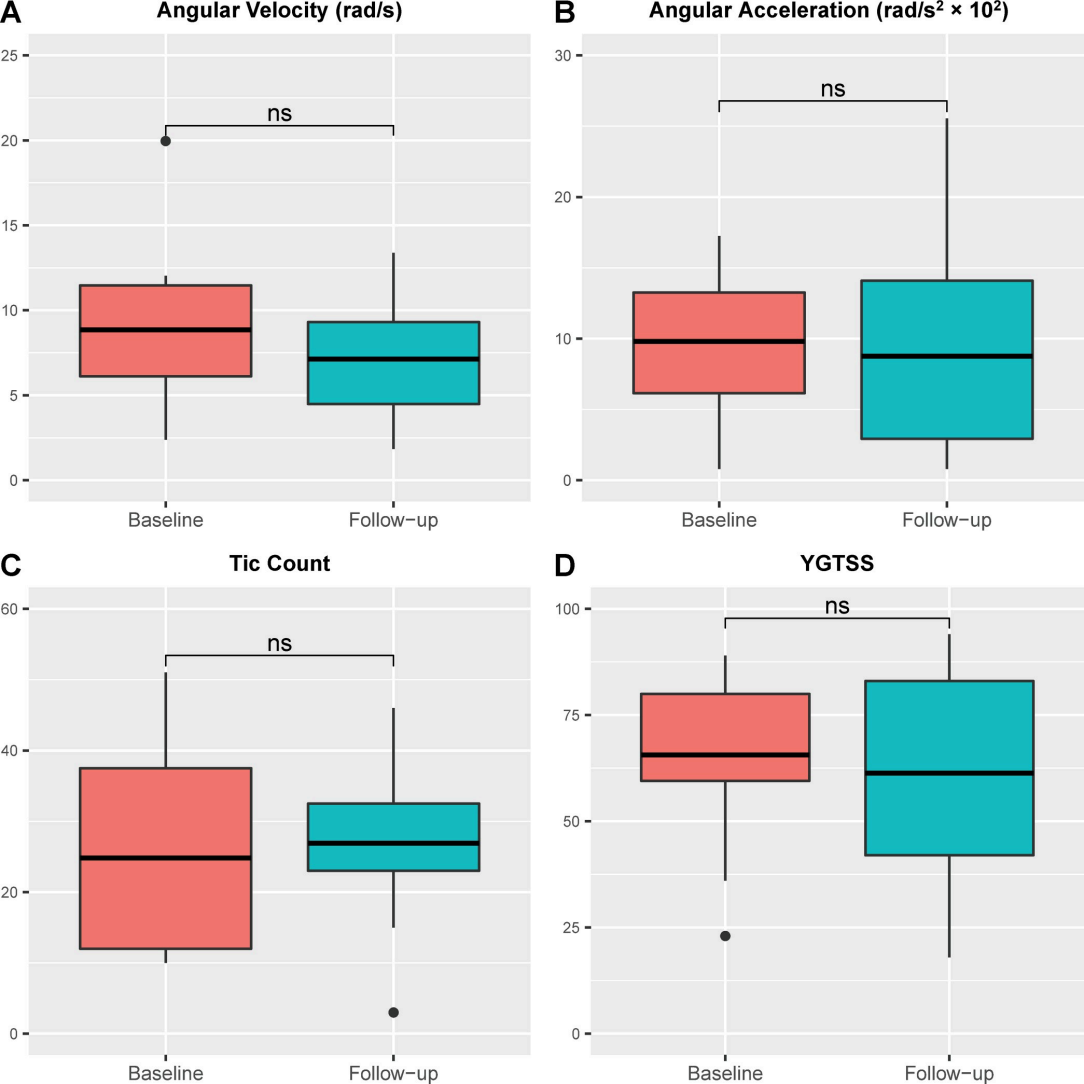
Group level comparison between baseline and 2-year follow-up values. Box-and-whisker plot showing mean values, interquartile ranges (IQR) (boxes) and 1.5×IQR (whiskers). A: Angular velocity. B: Angular acceleration. C: Tic count during a 5-minute experimental period. D: Yale Global Tic Severity Scale (YGTSS) score.

No significant change was observed between baseline and follow-up. Angular velocity at follow-up (median, 7.84 [IQR: 4.48–9.31] rad/s) did not differ from that at baseline (7.22 [6.12–11.47] rad/s) (P = 0.44), nor did the angular acceleration (533.14 [292.45–1409.36] rad/s^2^ vs. 1135.77 [613.76–1326.03] rad/s^2^) (P = 0.75) (Fig 2A and 2B). Tic count at follow-up (30 [23–32.5]) showed no significant change from that at baseline (17 [12–37.5]) (P = 0.67) (Fig 2C). The YGTSS score at follow-up (60 [42–83]) did not differ from that at baseline (70 [59.5–80]) (P = 0.82) (Fig 2D).

**Fig 3:**
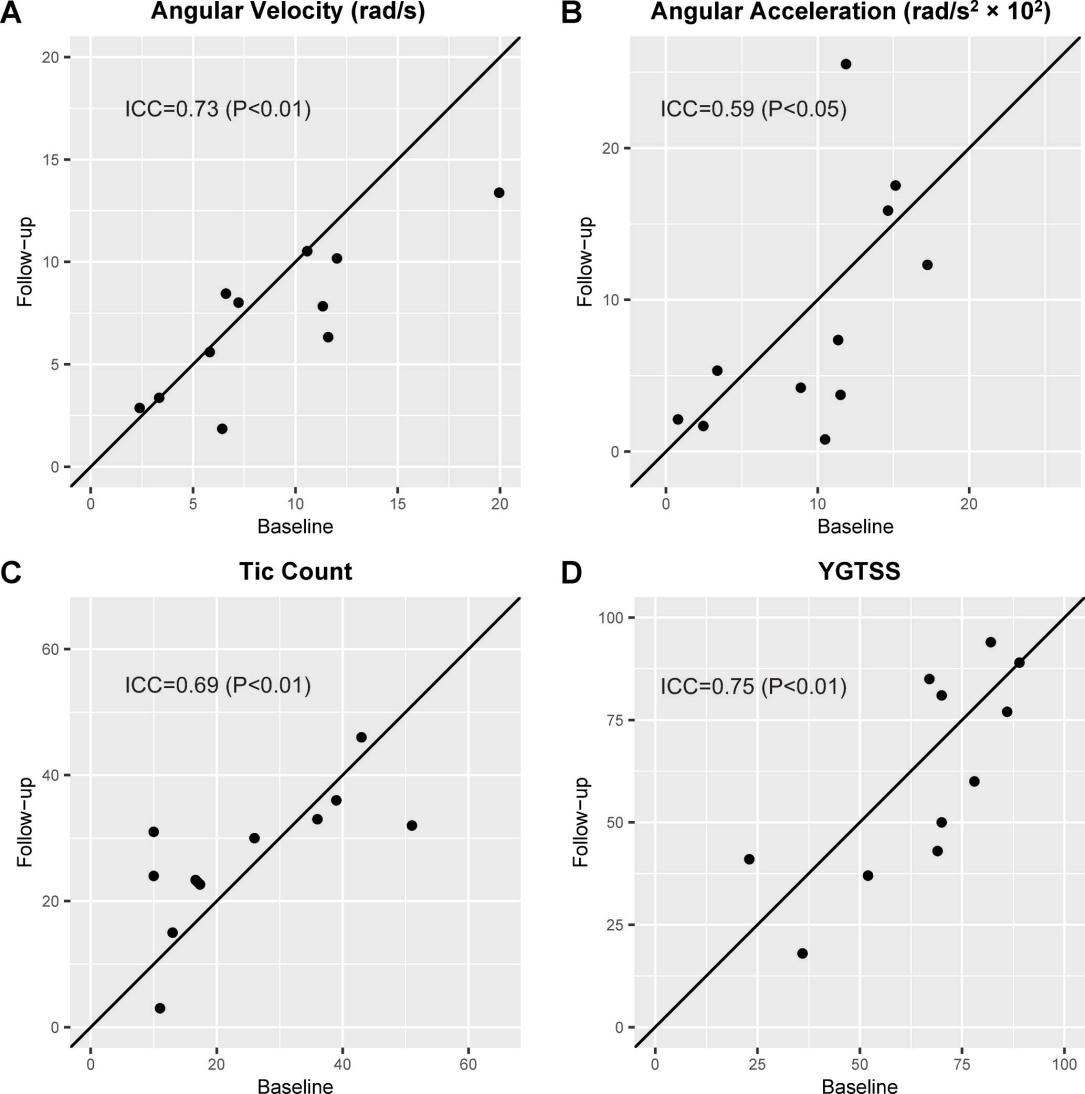
Individual level consistency between baseline and 2-year follow-up values. A: Angular velocity. B: Angular acceleration. C: Tic count during a 5-minute experimental period. D: Yale Global Tic Severity Scale (YGTSS) score. The solid line represents a perfect agreement of the two measurements.

At the individual level, ICC for angular velocity showed substantial consistency (0.73, 95% CI: 0.39–0.90, P = 0.0024) (Fig 3A). ICC for angular acceleration showed moderate consistency (0.59, 95% CI 0.15–0.84, P = 0.018) (Fig 3B). ICC for tic counts showed moderate consistency (0.69, 95% CI 0.32–0.88, P = 0.0047) (Fig 3C). ICC for YGTSS score showed substantial consistency (0.75, 95% CI 0.42–0.91, P = 0.0018). (Fig 3D).

## Discussion

This study was designed to elucidate the clinical course of neck tics at the group and individual levels. Analysis at the group level indicated no significant changes in angular velocity and acceleration of neck tics, tic counts, and YGTSS scores from baseline to follow-up. Previous studies that investigated group level changes in tics have shown that tic symptoms tend to alleviate during the clinical course at their pre or early teens in roughly three-quarters of children and over one-third of patients achieved remission [1, 2]. Nevertheless, other studies report that some patients did not achieve remission and continued to manifest tics as adult TS patients [20, 21]. The previous study reported that one out of 36 TS patients continued to have marked level of tic severity when he/she was 18 years of age [22]. Therefore, we hypothesize that because the participants in this study were older, they were less likely to exhibit alleviated symptoms or achieve remission, and manifested tics in a consistent manner.

Analysis at the individual level showed substantial consistency between baseline and follow-up for angular velocity and YGTSS scores; for angular acceleration and tic counts, moderate consistency was observed. Our finding supported the previous report that tic scores at the baseline predicted the scores at the follow-up at an interval of 6 years [1].

The force applied to a head or a neck can be estimated using angular velocity and angular acceleration. Therefore, the following are correlated: angular velocity/acceleration and impact to the head or neck; impact frequency and damage to the head or neck [23, 24]. Consequently, patients with swift or frequent neck tics continue to have high impact to their necks. Even though the exact mechanism of severe neck complications remains unknown, forceful repetitive neck tics are speculated to be a prominent risk factor [25]. Therefore, our findings underscored the importance of repeated measurement of neck tics to evaluate the risk of severe neck complications.

In the baseline data, angular velocity and tic counts were higher in participants of this study than in those who did not participate in the present study. This indicates that patients with severe neck tics are more likely to continue follow-up visits than patients with milder neck tics. We speculate that patients with milder neck tics followed a more typical course, in which the neck tics are more attenuated in intensity and frequency [1, 2]. Therefore, they did not continue regular visits as opposed to those with more intense and frequent neck tics at baseline. In this case, the participants in this study are patients with refractory neck tics, and our findings may not be applicable to the general TS population. Nevertheless, our findings suggest that a subgroup of patients, i.e., those with intense or frequent neck tics or considered to be refractory, would benefit from regular follow-ups and measurement using gyroscopes. They should be closely monitored in collaboration with movement disorder specialists to counteract any injuries resulting from neck tics. Furthermore, because this group is refractory to conventional therapeutic approach, they could also benefit from nonconventional treatment approaches such as D1 receptor antagonist, cannabis-based medications, transcranial magnetic stimulation, botulinum toxin injections, and deep brain stimulation [26].

Our findings indicate that the values obtained for these parameters at a given time can predict those in the future. In other words, patients with high-velocity neck tics will continue to have high-velocity neck tics, and patients with frequent neck tics will continue having frequent neck tics. Moreover, high YGTSS scores will remain high.

Our results are thought to have many confounders including effect of treatment, age, comorbidity, and duration of follow-up. Because the pharmacotherapy for the participants in this study did not drastically change between two time points, and none of the participants received intensive cognitive behavioral therapy. Therefore, we concluded that the treatment effects were unlikely to confound the results of the present study, but other factors might change the clinical course and thereby influence the results.

Our method requires manual annotation for neck tics. For example, an emerging method called behavior imaging enables completely automated behavioral annotation using two-dimensional (2D), three-dimensional (3D) sensors and 3D reconstruction technique [27]. Before this study, we planned to construct auto-detection system for motor tics using a motion sensor (Kinect for Windows. Microsoft. USA). We found that the sampling rate (30 Hz) of the motion sensor was insufficient for the detection of swift tics, and some motor tics involve slow motion and are difficult to distinguish from voluntary movements. Therefore, we concluded that it is extremely difficult to construct an auto-detection system for motor tics using a motion sensor. During recording sessions, we had noticed that patients with TS tended to rotate their necks extremely swiftly. Consequently, we changed our treatment plan and started our investigation for neck tics instead. Manual annotation of neck tics is time consuming, so our method needs refinement.

This study has some limitations. First, detailed clinical evaluation and experiments could not be performed for those who refused to participate or were lost to follow-up in the present study. In addition, as discussed above, because those with milder neck tics were considered to have been lost to follow-up, the participants in the present study might not represent the general TS population but TS population with refractory neck tics.

Therefore, with this additional data, our findings might change. Second, some of the patients with TS in our previous study manifested neck tics outside of but not within the clinical setting. Hence, the evaluation procedure described herein would not benefit them. Third, we evaluated neck tics only when the participants were sitting but not while they were standing or walking. Fourth, a 2-year interval may not be sufficient to detect chronological changes at group or individual levels. In addition, we evaluated only two timepoints of data, and this may be insufficient to demonstrate the clinical course. Fifth, the 5-min observation period may have been insufficient for definitive conclusions, since the small sample size limited the statistical power. Although we carried out rotational plane wise analysis in our previous study, the same was not performed in the current study because of the small sample size. Sixth, forehead IMU might influence tic frequency or severity. Seventh, because we did not assess the participants for neurological complications, it is difficult to draw a definitive conclusion.

## Conclusion

To our best knowledge, our study is the first quantitative follow-up study of neck tics in patients with TS. We found positive baseline/follow-up correlations for angular velocity, tic count, and YGTSS score. Although tic symptoms generally diminish during the clinical course, for patients with swift or frequent neck tics, such tics could continue to manifest. Despite its limitations, our study will facilitate the identification of TS patients at high risk for developing severe neck complications. It may also be useful in terms of differential diagnosis, to have a comprehensive evaluation of therapeutic efficacy, and elucidation of TS pathophysiology. Further longitudinal studies with larger sample sizes and longer and shorter experimental intervals are needed to fully clarify the clinical course of neck tics, in order to eventually progress towards preventing severe neck complications.

## Supporting information

S1 Table Details and experimental results on the participants of present study. (XLSX)
